# Sex-chromosome evolution in frogs: what role for sex-antagonistic genes?

**DOI:** 10.1098/rstb.2020.0094

**Published:** 2021-08-30

**Authors:** Nicolas Perrin

**Affiliations:** Department of Ecology and Evolution, University of Lausanne, 1015 Lausanne, Switzerland

**Keywords:** amphibians, sex determination, sexual dimorphism, sex reversal

## Abstract

Sex-antagonistic (SA) genes are widely considered to be crucial players in the evolution of sex chromosomes, being instrumental in the arrest of recombination and degeneration of Y chromosomes, as well as important drivers of sex-chromosome turnovers. To test such claims, one needs to focus on systems at the early stages of differentiation, ideally with a high turnover rate. Here, I review recent work on two families of amphibians, Ranidae (true frogs) and Hylidae (tree frogs), to show that results gathered so far from these groups provide no support for a significant role of SA genes in the evolutionary dynamics of their sex chromosomes. The findings support instead a central role for neutral processes and deleterious mutations.

This article is part of the theme issue ‘Challenging the paradigm in sex chromosome evolution: empirical and theoretical insights with a focus on vertebrates (Part I)’.

## 1. Introduction

Most mammals and birds, as well as many insects such as *Drosophila*, present highly heteromorphic sex chromosomes, with a small and gene-poor Y (or W) chromosome contrasting with a large and gene-rich X (or Z) chromosome. The so-called canonical model of sex-chromosome evolution, conceived to account for these patterns, assigns an instrumental role to sex-antagonistic (SA) genes in the process of degeneration. According to this model, a sex-determining (SD) mutation newly fixed on a chromosome (such that individuals with the mutation develop into one sex, and individuals without it into the other sex) will automatically favour SA mutations occurring in its vicinity: if linked to a male-determining allele, for instance, a male-beneficial mutation will spread even if highly detrimental to females, because linkage makes it more likely to be transmitted to sons than to daughters. Then, mutations that further restrict or arrest X–Y recombination between the SD and SA genes (e.g. an inversion) will also spread, because the recombination load will be thereby alleviated or eliminated. As a side effect of recombination arrest, however, the Y (or W) chromosome will start to accumulate deleterious mutations, and progressively degenerate [1–7].

Along the same logic, SA genes have also been proposed to play a key role in driving Y-autosome fusions [8] and sex-chromosome turnover [9,10], by just reversing the model above: the spread of a male-determining mutation will be favoured by linkage to a male-beneficial allele, because linkage makes the male-beneficial/female-detrimental allele more likely to be transmitted to sons than to daughters.

Though elegant and intellectually appealing, the canonical model has received limited empirical support. It cannot be tested in systems with differentiated sex chromosomes (for which it was developed), because SA genes on these chromosomes might have accumulated after recombination has arrested, or after turnovers have occurred. For a proper test, one needs to focus on ongoing turnovers or systems at incipient stages of differentiation. Frogs are ideal systems in this context. Their sex chromosomes are still morphologically undifferentiated, but show polymorphism in the level of genetic differentiation (i.e. in the frequency of XY recombination); they also undergo frequent transitions, some of which are still ongoing in some species (where sex chromosomes differ between populations). Here, I review some work performed in this context, mostly over the last decade, on European species of frogs from two families, Ranidae (true frogs) and Hylidae (tree frogs), with a special focus on the European common frog, *Rana temporaria*.

## 2. Genetic sex determination, homomorphic sex chromosomes and male heterogamety

All species of frogs properly investigated so far have revealed a genetic component to sex determination (GSD), even if genetic control is not always strict [11,12]. Some laboratory studies have suggested a masculinizing effect of high temperatures, but at values (27–36°C) that largely exceed those prevailing during larval development [13]. Thus, there is no direct evidence for environmental effects on sex determination under natural settings, and GSD normally prevails in nature. Surprisingly, however, sex chromosomes have remained morphologically undifferentiated (i.e. homomorphic) in more than 96% of species [12]. Thus, the existence of GSD and the patterns of heterogamety have been usually established, not by karyotype analyses (with a few exceptions; e.g. [14]), but via experimental gynogenesis (gynogenetic individuals are all females in XY systems), sex reversals (sex-reversed XX males have all-female progenies) or genetic markers (sex-linked markers in XY systems preferentially transmit one paternal allele to sons and the other to daughters).

The first data along this latter line were gathered from enzymatic polymorphisms (see [15] for a review). Surprisingly, most species investigated in these early studies turned out to be male heterogametic (XY males; [15,16]). The prevalence of XY systems across both Hylidae and Ranidae has been largely confirmed, with the use of more powerful molecular tools such as microsatellites (e.g. [17]) or RADseq [18]. Male heterogamety actually prevails among amphibians in general, comprising two thirds (68 of 102) of the species for which heterogamety has been identified so far [19].

## 3. Restricted male recombination

Interestingly, searches for sex-linked markers in XY species have typically unveiled large numbers of male-specific markers. A high-density sex-specific linkage map established from a *Hyla arborea* family, for instance, revealed a threefold increase in single nucleotide polymorphism (SNP) density in the male relative to the female map for chromosome 1 (the sex chromosome) [18]. This clearly suggests that, even though sex chromosomes are not morphologically differentiated, X and Y chromosomes have stopped recombining over a large segment for a significant number of generations.

**Figure 1.**
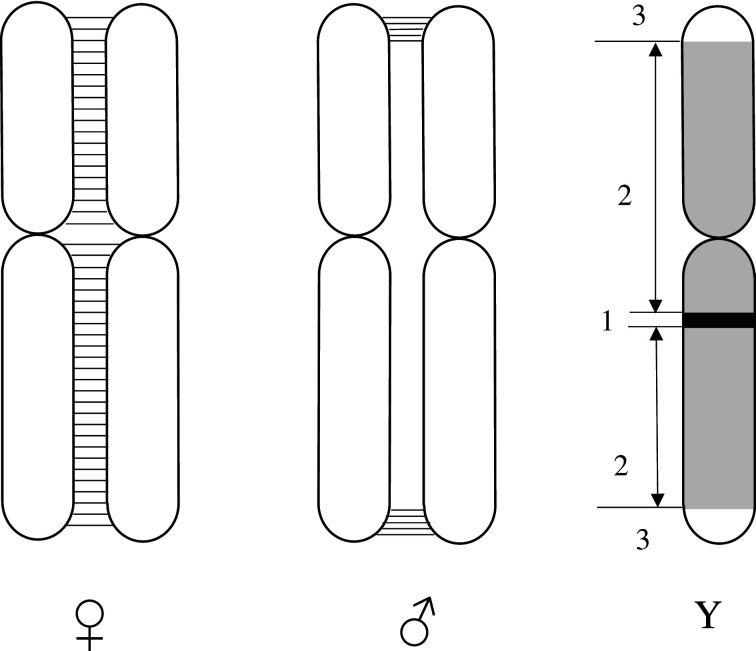
Sex-specific patterns of recombination in frogs. Females (left) recombine more or less uniformly all along their chromosomes genome wide, while males (centre) recombine mostly or only at chromosome tips (where crossovers are more frequent than in females). This implies that, under strict GSD, Y chromosomes (right) will show differentiation all over except for the tips (strata 1 and 2), but only at stratum 1 (the SD region) under leaky GSD. All three strata remain undifferentiated under non-GSD.

That recombination is suppressed or restricted in male frogs had already been documented with enzymatic markers (e.g. [15]). This pattern has been largely confirmed, using more powerful molecular tools (e.g. [20,21]). Importantly, it is not limited to sex chromosomes: high-density linkage maps typically find shorter maps in males than in females for all chromosomes, with a characteristic central peak in SNP density corresponding to a large non-recombining segment (Hylidae: [18]; Ranidae: [22]). Thus, recombination occurs uniformly across chromosomes in females, but mostly at chromosome tips in males (where recombination rate actually exceeds that in females; figure 1; also see fig. S10 in [23]). These results are in line with cytological evidence that, for anurans in general (except for the early branching Leiopelmatoidea and Discoglossoidea), male meiosis presents two and only two chiasmata per bivalent, which are always terminal, giving them a typical ring shape during metaphase I [24]. Similar patterns have been documented in fishes (sticklebacks: [25]; fugu: [26]; guppies: [27,28]) and reptiles [29]. More generally, most vertebrates and many other eukaryotes show a recombination bias toward telomeres in males, and more uniformly spread in females (see [30] for a documentation of patterns and thorough discussion of possible evolutionary causes and consequences).

Given that autosomes do recombine in females, their arrest of recombination in males does not result from structural changes (such as inversions), but more likely from some specificities of the male meiosis. It is tempting to extrapolate this conclusion to sex chromosomes, namely that the arrest of X–Y recombination in frogs does not stem from structural changes (as classically assumed by the canonical model), but only from the fact that Y chromosomes are found in males, in which recombination only occurs at chromosome tips. This conjecture is fully confirmed by observations of naturally occurring sex reversals: X–X recombination is stopped in XX males, while X and Y fully recombine in XY females [31]. Thus, recombination patterns in general (both on autosomes and on sex chromosomes) are controlled by phenotypic sex, not by genotypic sex. The arrest of XY recombination in frogs is, therefore, a direct and necessary consequence of male heterogamety. Any chromosome that takes an SD role and becomes strictly male-limited stops recombining *ipso facto*. There is no need to invoke a role for SA genes in this process.

## 4. Leaky genetic sex determination and sex reversal

Sex reversals, i.e. discrepancies between genetic and phenotypic sex, appear widespread across frog populations. XY females, however, seem much rarer than XX males. Interestingly, the frequency of sex reversals varies among populations, as largely documented in *R. temporaria* [32–34], where this variation seemingly relates to the phylogeographic origins of populations, not to abiotic factors such as temperature (see below). At one end of the continuum are populations with strict GSD, such as those found in northern Sweden and the southern Swiss Alps, where offspring sex is strictly controlled by the paternally inherited copy of chromosome 1 (which also acts as the sex chromosome in this species). Other populations, such as those found in southern Sweden and the northern Swiss Alps, display ‘leaky GSD’: offspring sex correlates significantly, but not strictly, with the paternally inherited copy of chromosome 1. At the other end are populations, such as those found in lowland Switzerland and Alsace [21,22], that show no sign of a genetic component to sex determination (non-GSD): offspring sex does not correlate with the paternally or maternally inherited copy of any chromosome pair or genetic marker.

Accordingly, these populations also differ in the level of sex-chromosome differentiation. A meaningful distinction is to be made here between the three sex-chromosome strata (figure 1). A first one comprises the immediate surrounding of the SD locus (the best candidate in *R. temporaria* being *Dmrt1*, a transcription factor known to play a key role in sex determination and sexual development across all metazoans), which is expected to differ between sexes if this locus is to determine sex. A second stratum is made of the largest, central part of sex chromosomes (comprising the bulk of sex-linked genes), which does not recombine in males; this part is expected to show some sex differentiation under strict GSD (because the Y chromosome then only occurs in males). The third stratum, finally, comprises the two tips of chromosomes, which recombine in males and are, therefore, never expected to show sex differentiation.

Males sampled from a series of populations were tested for (i) markers located within the candidate SD segment (namely, three markers within introns 1, 2 and 5 of *Dmrt1* and one in the first intron of the closest downstream gene, *Dmrt3*; no polymorphism was found within exons of these genes), and (ii) series of anonymous microsatellite markers along chromosome 1, encompassing the second and third strata defined above [35,36]. According to the above expectations, males of families showing strict GSD display XY differentiation over their whole chromosome 1, except for the tips (i.e. strata 1 and 2 are sex-differentiated). These males are referred to as XY males. Males of families with leaky GSD, by contrast, only differ from females at *Dmrt* markers (stratum 1); these Y chromosomes are referred to as ‘proto-Y chromosomes’, and their carriers as XY° males. Finally, males of non-GSD families do not show any differentiation from females, even at the *Dmrt* markers. These males, which seem genetically identical to females, are referred to as XX males.

Similar polymorphisms in sex-determination patterns are likely widespread across natural populations of other frogs. Sex reversals and leaky GSD are now being documented in the several species for which sex-linked markers have been developed (e.g. *Rana clamitans*, [37]; *Rana dalmatina*, [38]). Additionally, Jeffries *et al*. [23] found polymorphism in Y-haplotypes, in the levels of Y chromosome differentiation, as well as populations in which no sex-linked marker could be found in six species of Ranidae. Occasional X–Y recombination has also been inferred from the patterns of sex-chromosome evolution in Hylid frogs [39,40]. All are hallmarks of leaky sex determination. However, the geographical distributions of these polymorphisms remain to be investigated.

## 5. Threshold model of sex determination

**Figure 2.**
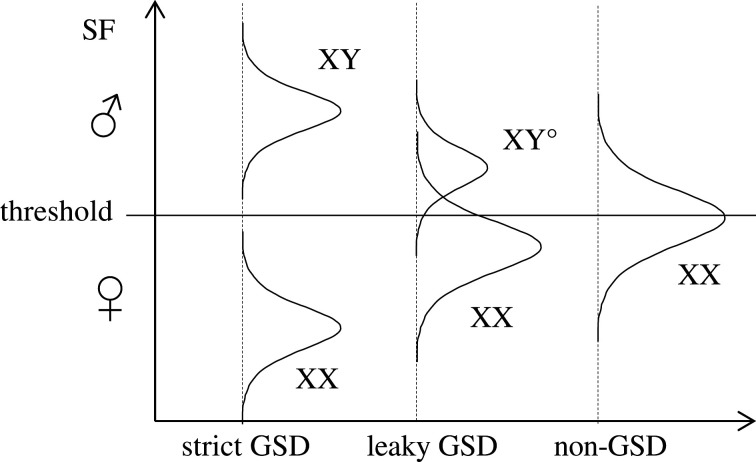
Threshold model of sex determination. An individual develops as male if the expression of the sex factor (SF) exceeds a given threshold, and as female otherwise. Strict GSD results if all XY individuals, but none of the XX, exceed the threshold (left). Leaky GSD results if XY and XX distributions overlap somewhat (centre). Non-GSD results if a single genotype (XX) occurs; individual sex is then determined by random noise in the expression of the sex factor (right).

The above patterns fit the so-called ‘threshold model’ of sex determination (figure 2), according to which sex is determined by the expression level of a sex factor (SF, which might be *Dmrt1* in *R. temporaria*) during a sensitive period of development. An individual develops into one sex (let us say male) if the expression exceeds a given threshold, and into the other sex if that threshold is not met. Assuming that the Y copy of the SF gene is expressed much more than the X copy, all XY individuals should lie above the threshold (and thus develop as males), and all XX individuals below the threshold (and thus develop as females), resulting in strict GSD. If expression levels overlap somewhat, then random variation makes some XX individuals develop as males, and some XY individuals as females, resulting in leaky GSD. Given that X and Y recombine in females, these occasional events of sex reversal will prevent sex-chromosome differentiation, except in the immediate vicinity of the SD locus, resulting in XY° males with proto-sex chromosomes (the fountain-of-youth model; [41]). Finally, if the two copies show no statistical difference in expression level, then individual sex is determined by random noise in the expression of the SF [42], resulting in XX males and non-GSD.

In line with this model, XY and XY° *R. temporaria* males typically present different Y-specific *Dmrt1* alleles (see below), while XX males by definition share the same *Dmrt1* alleles as females. Thus, the observed polymorphism of sex-chromosome differentiation in common frogs seems best explained (proximate cause) by a polymorphism at the SD locus, where different alleles vary in their degree of penetrance: the more penetrant a masculinizing allele is (i.e. the more likely bearers of this allele develop as males), the less frequently X and Y recombine, and the more differentiated sex chromosomes are.

## 6. A role for phylogeography

As just mentioned, the relative frequencies of XY, XY° and XX males within *R. temporaria* populations strongly covary with the Y-specific alleles fixed at the *Dmrt1* markers. Five main Y-specific *Dmrt1* haplogroups (labelled Y_A_, Y_B_, Y_C_, Y_D_ and Y_E_) have been identified so far across the species range (which spans south–north from Spain to Norway, and east–west from Russia to Ireland). Haplogroup distributions closely correspond to those of the main mtDNA haplotypes documented in this species (e.g. [43–45]), pointing to a key role of phylogeography (i.e. historical range expansions from glacial refugia) in their present-day distribution. In Switzerland, for instance, haplogroup Y_A_ is found south of the main Alpine range, in association with the mtDNA Alpine sublineage I mostly spread in Italy ([43]; CH-South in [45]), and haplogroup Y_B_ north of this range, in association with the mtDNA Alpine sublineage III ([43]; CH-North in [45]). Males from Y_A_ populations tend to display differentiated Y chromosomes associated with strict GSD (XY males), while those from Y_B_ populations typically have proto-sex chromosomes with leaky GSD (XY° males), or undifferentiated chromosomes with non-GSD (XX males). Despite the high range of elevations investigated (325 m to 2655 m a.s.l.), elevation only plays a marginal role on sex-chromosome differentiation in Y_B_ populations, and none in Y_A_ populations [34]. Haplogroup Y_B_ is spread throughout most of western Europe up to southern Sweden (e.g. in Tvedöra), where it also associates with XY° and XX males. Further north (e.g. in Ammarnäs) and throughout eastern Europe occurs the haplogroup Y_C_ (co-distributed with the main eastern mtDNA haplogroup T5; [44]), mostly associated with XY males (strict GSD and differentiated sex chromosomes). Importantly, these associations also hold within populations at contact zones, where both haplogroups coexist [36]. Hence, sex-chromosome differentiation (and penetrance of SD alleles) bears a clear link with phylogeography, not with climate or any other environmental feature. A similar situation was documented in *Rana iberica* [23] and *H. arborea* (for which *Dmrt1* is also the candidate SD gene; [46]), where sex-chromosome differentiation parallels range expansion from glacial refugia [47].

## 7. No evidence that differentiated Y chromosomes affect male (or female) fitness

One basic tenet from the canonical model is that sex-chromosome differentiation associates with the fixation of SA genes. Common frogs offer a unique opportunity to test this assumption, given that XX, XY° and XY males sometimes coexist within the same populations. One obvious prediction from this model would be that XY males are better than XX males at mating with females and/or siring clutches, thanks to sexually dimorphic traits particularly attractive to females. Note that, to allow coexistence, this fitness benefit should be balanced by some negative consequence of sex-chromosome differentiation, such as decreased survival owing to the accumulation of deleterious mutations on the non-recombining segment.

Morphological measurements of more than 800 XY, XY° and XX males within one population from the western Swiss Alps failed to find any phenotypic difference between these categories of males [48]. Despite a marked sexual dimorphism (sexes differ in size, colour and body proportions), no morphological trait differed significantly between categories; a male is a male, whatever the state of differentiation of its sex chromosomes. Similarly, the probabilities of successful mating and of siring a clutch did not differ between XY, XY° and XX males. Along the same line, XY females seem also perfectly viable and fertile [31], which argues against the fixation of male-beneficial/female-detrimental alleles on the Y. All of this strongly suggests that sexual dimorphism entirely or at least predominantly results from the differential expression of autosomal genes, not from the fixation of sex-limited genes on sex chromosomes.

Thus, SA genes do not seem to play a significant role in the early steps of sex-chromosome evolution in frogs. This conjecture is supported by comparisons of the transcriptomes of XY, XY° and XX males to those of XX females: despite pervasive sex biases in the expression of many genes, all males present the same profiles, independent of sex-chromosome differentiation. Chromosome 1 in XY males, moreover, does not harbour more sex-biased genes than autosomes [49,50].

## 8. Are sex chromosomes a good location for sex-antagonistic genes?

These empirical results are actually backed by theoretical approaches. Individual-based simulations were performed to investigate the evolution of XY recombination, under the opposing forces of SA selection (which selects against recombination) and deleterious mutations (which select for recombination) [51,52]. These simulations show that, depending on their rates, mildly deleterious mutations have indeed the potential to oppose SA selection and select for a low equilibrium level of XY recombination (mediated e.g. by sex reversal). The resulting rare occurrence of XY females (in the order of one per population every three to four generations, intriguingly close to the rate of XY recombination estimated by Guerrero *et al*. [40] for Hylid frogs) seems sufficient to largely purge the load of deleterious mutations from the Y. Note that X–Y recombination actually benefits males, not females (the accumulation of deleterious mutations on non-recombining Y chromosomes lowers male survival, but boosts their purge from the X, which increases female survival). These rare recombination events oppose the fixation of SA mutations on the Y, owing to recombination load (since male-beneficial/female-detrimental alleles would then be transmitted to the X). Fixation is also impeded by Hill–Robertson interferences with deleterious mutations. Altogether, these simulations suggest that sex chromosomes might not be a good location for SA genes, and sex conflicts better solved through the differential expression of autosomal genes.

## 9. A role for neutral forces?

These results raise the question of what evolutionary causes might favour strict versus leaky GSD, and more generally maintain the polymorphism in X–Y recombination and sex-chromosome differentiation observed in frogs. Stronger sex-ratio selection in small populations might play a role, as suggested by the association of strict GSD with post-glacial expansions, documented in *H. arborea* and *R. iberica*. However, with the data in hand, one cannot exclude the idea that such polymorphism only results from neutral genetic drift, whereby SD alleles with different levels of penetrance were fixed in small populations inhabiting different glacial refugia. If, by chance, the allele fixed had low penetrance (such as for haplogroup Y_B_), a leaky GSD will result, and sex chromosomes are expected to remain morphologically undifferentiated. If a stronger-penetrance allele was fixed (such as for haplogroups Y_A_ and Y_C_), then a stricter GSD will result; sex chromosomes are expected to progressively differentiate, and thus the Y to progressively accumulate deleterious mutations. At some level, the fitness of these Y chromosomes might decrease to such a point that a sex-chromosome turnover is expected.

## 10. Sex-chromosome turnovers

Early work in Ranidae using isozymes had found that sex is associated with different linkage groups in different species, or even different populations of the same species, pointing to a labile position of the sex locus [15,53]. As already mentioned, isozyme inheritance patterns had also unveiled widespread male heterogamety. Both patterns have been formally tested and fully confirmed with an expanded dataset using RADseq approaches [23]. Despite a high rate of sex-chromosome turnover, all of the 24 species of Ranidae investigated for which heterogamety could be identified display an XY system, with the exception of *Glandirana rugosa*, where both XY and ZW populations have been found across different races in Japan [54].

Similar patterns were documented from Hylidae, where male heterogamety prevails despite high rates of turnover [55]. Only two transitions towards female heterogamety have been documented in this family. One of them occurred more than 11 Ma in the lineage leading to *Hyla sarda* and *Hyla savignyi* [56]. Interestingly, despite being female-heterogametic for millions of years, both species have conserved the typical pattern of heterochiasmy (figure 1). Thus, sex chromosomes recombine in ZW females, not in ZZ males. This strongly supports the idea that the drastic heterochiasmy documented in anurans results from intrinsic constraints on male meiosis, and is neither the cause nor the consequence of male heterogamety. Furthermore, despite their high rate of ZW recombination (which prevents the fixation of SA genes on the W), both species display the same level of sexual dimorphism as other Hylidae [57], which adds to the growing evidence that sexual dimorphism in frogs results from the differential expression of autosomal genes, not from the sex linkage of sex-specific genes.

Four main classes of ultimate causes are considered to have the potential to drive sex-chromosome turnovers [58]: (i) neutral genetic drift, (ii) sex-ratio selection, (iii) SA selection, and (iv) selection stemming from the accumulation of deleterious mutations. Importantly, these potential causes make different predictions regarding both the recurrence of transitions and the changes in patterns of heterogamety during turnovers.

(i) Transitions mediated by *genetic drift* [59] were recently investigated via evolutionary modelling [60–62], with a focus on epistatically dominant SD mutations (meaning that XX individuals with a masculinizing mutation M are males (XXmM), and XY individuals with a feminizing mutation F are females (XYfF)). It appears from these simulations that a transition which replaces an old Y (or W) chromosome by a new one (i.e. that maintains the patterns of heterogamety) is about four times more likely than the fixation of a neutral autosomal mutation, because the new sex chromosome has to reach a frequency of 0.25, not 1.00. For the same reason, such transitions are also two to four times more likely than those that change heterogamety (e.g. changes from XY to ZW), because in such transitions the old Y is fixed as an autosome (resulting in YYff males and YYfF females), so that its frequency has to rise from 0.25 to 1.00. The likelihood for this latter kind of transitions increases as effective population size (N_e_) decreases. This differs from the dynamics of classical neutral mutations (the fixation of which only depends on mutation rate; [63]), because random changes in allele frequencies at the sex locus affect population sex ratio, which accelerates the fixation of a dominant SD mutation (drift-induced selection). Thus, depending on N_e_, one out of 2–5 transitions occurring under genetic drift is expected to change the patterns of heterogamety, a frequency markedly higher than that documented in Ranidae.
(ii) Transitions may also be driven by *sex-ratio biases* stemming from e.g. meiotic drive [64] or environmental factors such as climatic change [65] or parasites [66]. Sex-ratio selection is probably responsible for the only exception to XY sex determination in the Ranidae dataset analysed by Jeffries *et al*. [23]. The ZW races of *G. rugosa* were shown to stem from crosses between two highly divergent XY races; experimental crosses between these same races produce a male-biased progeny, which is expected to favour the spread of epistatically dominant feminizing mutations [54]. In general, however, male- or female biases should occur *a priori* with equal probability, so that turnovers triggered by this selective pressure should maintain or change heterogamety with equal probability.
(iii) The rationale underlying *SA-driven turnovers* [9,10] was presented in the Introduction. Whether these turnovers change the pattern of heterogamety or not similarly depends on whether the newly arising SA mutation is male- or female-beneficial. These two kinds of mutations have *a priori* equal probability. In the case of frogs, however, heterochiasmy (drastically reduced male recombination) might facilitate transitions to XY systems, owing to the immediate linkage it creates between male-determining and male-beneficial genes. By the same logic, SA selection is unlikely to have contributed to the few XY-to-ZW transitions documented in Ranidae and Hylidae: the high rate of female recombination impedes the establishment of linkage between female-determining and female-beneficial genes on the W. Although SA selection might in principle favour an XY-to-XY transition in frogs, it is unlikely to trigger the kind of continuous turnover that characterizes Ranidae and Hylidae: once fixed on the new sex chromosome after a first transition, a male-beneficial mutation should strongly oppose further changes, the more so that, following the first transition, both SA effects and SA–SD linkage are expected to rapidly strengthen [61,67,68].
(iv) By contrast, the load of *deleterious mutations* accumulating on non-recombining genomic regions has the potential to drive this sort of cyclic turnover (the ‘hot-potato model’; [67,68]). As soon as a fully penetrant male-determining mutation is fixed, the entire Y chromosome stops recombining (except for the tips); Hill–Robertson interferences involving hundreds or thousands of genes facilitate the rapid accumulation of deleterious mutations and decay of the new Y, decreasing its fitness until a new turnover becomes unavoidable (sex determination literally ‘burns the hands’ of the chromosome in charge). Such turnovers, furthermore, are expected to be biased towards the maintenance of heterogamety. Provided the new masculinizing mutation M is dominant (i.e. XXmM are males), then the old and decayed Y is discarded (which is exactly what triggers the transition). A dominant feminizing mutation F, by contrast, leads to a female-heterogametic system (YYff males and YYfF females), during which the Y is fixed as an autosome. This outcome is of course strongly counter-selected if the Y is loaded with deleterious mutations. The only way to change heterogamety would be through a recessive masculinizing mutation M generating XXmM females and XXMM males [59], which would fix the X and eliminate the loaded Y. This sort of transition is much less likely, however, because the mutation is not visible to selection until it has spread (by drift) to frequencies high enough to produce homozygotes. Hence, the patterns documented in frogs are compatible with a role for deleterious mutations, assuming SD mutations are generally dominant.

Altogether, therefore, the combination of high turnover rate and maintenance of male heterogamety suggests a central role for the accumulation of deleterious mutations on non-recombining genomic regions as a driver of sex-chromosome transitions, rather than for SA selection.

## 11. Summary and conclusion

The canonical model of sex-chromosome evolution, which assigns a crucial role to SA genes, has received wide acceptance, and is systematically invoked to account for the arrest of recombination and ensuing degeneration that characterize the fully differentiated sex chromosomes currently found in mammals, birds and insects. Although elegant and appealing, this model relies partly on verbal arguments, some of which are opposed by individual-based simulations [51,52]. From these simulations, deleterious mutations accumulating on non-recombining chromosomes have the potential to oppose the fixation of SA genes on sex chromosomes. The latter might, therefore, not be the best location for genes that underlie sexual dimorphism. Several aspects of the canonical model (in particular the selective forces acting on and resulting from reduced levels of XY recombination) should be better formalized, and auxiliary assumptions clarified.

Empirical support, furthermore, is rather limited. Such support should optimally come from sex chromosomes at incipient and variable levels of differentiation. Guppies (*Poecilia reticulata*) might present an ideal model in this respect, owing to their strong sexual dimorphism and sex-linked polymorphism in male coloration [69]. Genomic analyses of three pairs of populations from Trinidad [70] suggested an instrumental role for SA genes, with three independent events of expansion of the non-recombining region in upstream populations, following changes in sexual selection stemming from a decrease in predation pressure. Further analyses, however, are casting doubts on this scenario, opposing the claim for independent evolutionary strata [27,71]. These analyses suggest instead the buildup of SA genes to be a consequence of pre-existing patterns of reduced male recombination genome wide.

In frogs, as the present review makes clear, the canonical model finds little support either. The dynamics of sex-chromosome differentiation do not seem to be significantly affected by SA genes, as supported by several lines of arguments.

(i) Like in guppies, there is no need to invoke SA genes to account for the arrest of XY recombination; this arrest is the direct consequence of male heterogamety combined with the strong heterochiasmy that characterizes most anurans. In female-heterogametic systems, ZZ males show the same patterns of drastically reduced recombination (so that sex chromosomes recombine in ZW females, not in ZZ males), suggesting this heterochiasmy results from constraints on male meiosis.
(ii) The geographical polymorphism in sex-chromosome differentiation correlates closely with phylogeography, not with environmental features or associated selective forces. This polymorphism results from the fixation, possibly by genetic drift in glacial-refugia populations, of SD alleles with different levels of penetrance.
(iii) Naturally occurring XX males display the same phenotype and mating success as XY males; XY females also seem perfectly functional and fertile, which argues against the fixation of male-beneficial/female-detrimental alleles on Y chromosomes.
(iv) Female-heterogametic species, in which Z and W recombine intensely, display the same level of sexual dimorphism as XY species, suggesting that sexual dimorphism does not rely on sex-limited genes.
(v) Transcriptome analyses unveil strong sex biases in gene expression, which however associate with phenotypic sex, not genetic sex; XX males show the same profiles as XY males, drastically different from XX females, confirming that sexual dimorphism essentially results from the differential expression of autosomal genes.
(vi) Genes on sex chromosomes show exactly the same levels of sex-biased expression as autosomal genes, supporting the above conclusion.
(vii) The patterns of sex-chromosome turnovers (recurrent cycles of transitions, combined with heavy biases towards the maintenance of male heterogamety), suggest they originate from the deleterious mutations accumulating on non-recombining genomic regions, rather than from SA genes.

The kind of investigations presented here should be expanded to other species and groups with incipient sex chromosomes. If the same patterns as documented in Ranidae and Hylidae also apply more widely, the inevitable conclusions will be that the role of SA genes in the early evolution of sex chromosomes has been overemphasized, and that we are now in need of alternative models to account for sex-chromosome evolution within a more general framework.
